# Impact of diagnosis on outcomes for compulsory treatment orders in New Zealand

**DOI:** 10.1192/bjo.2022.547

**Published:** 2022-08-01

**Authors:** Ben Beaglehole, Giles Newton-Howes, Richard Porter, Chris Frampton

**Affiliations:** Department of Psychological Medicine, University of Otago, Christchurch, New Zealand; Department of Psychological Medicine, University of Otago, Wellington, New Zealand

**Keywords:** Compulsory treatment, diagnosis, antipsychotics, coercion, psychotic disorders

## Abstract

**Background:**

Compulsory community treatment orders (CTOs) are controversial because they enforce psychiatric treatment of patients in the community. It is important to know which patients benefit from compulsory treatment to better inform CTO use.

**Aims:**

To examine the effect of a range of diagnoses on outcomes associated with CTOs to determine whether there are specific outcome signatures for CTOs according to diagnosis.

**Method:**

New Zealand's Ministry of Health databases provided demographic, service use and medication-dispensing data for all individuals placed on a CTO between 2009 and 2018. We used a hierarchical approach to categorise individuals according to diagnosis. Admission rates, admission days per year, community care and medication dispensing were analysed according to diagnosis and CTO status.

**Results:**

In total, 14 726 patients were placed on a CTO over the 10-year period between 1 January 2009 and 31 December 2018. For psychotic disorders, CTOs were associated with reduced admission frequency and duration. However, the opposite occurred for dementia disorders, bipolar disorders, major depressive disorder and personality disorders. Higher rates of medications, including depot antipsychotic medications, were dispensed on CTOs for all diagnostic groups.

**Conclusions:**

CTOs were associated with reduced admission frequency and admission days per year for patients with psychotic disorders, whereas the opposite occurred for other diagnostic groups. Rather than seeking to establish whether CTOs are effective, we suggest that there are specific outcome signatures associated with CTOs for different disorders and knowledge of these can improve understanding and clinical practice in this area.

Compulsory community treatment orders (CTOs) enable psychiatric treatment to occur in the community without the requirement for the patient's consent. A key goal of CTOs is to prevent readmission to psychiatric hospitals for people with severe mental illness and associated risks.^1^ CTOs are controversial because treatment is enforced during times when patients may be stable and free of symptoms. Three randomised controlled trials (RCTs) that evaluated CTOs^2,3,4^ were identified by a Cochrane review.^5^ Swartz et al concluded that individuals treated under a CTO of longer than 6 months duration had reduced hospital admissions and total hospital days when combined with intensive treatment.^6^ Steadman et al evaluated court-ordered treatment including enhanced services compared with an enhanced service package alone and reported that there were no differences between the two groups on all major outcome measures.^3^ The OCTET study randomised patients with psychosis to limited or extended compulsory community treatment and reported that the imposition of extended compulsory supervision did not reduce the rate of subsequent readmission.^4^ However, the Cochrane review concluded that the quality of RCT evidence evaluating CTOs was low to moderate and that CTOs did not result in clear differences for the majority of outcomes considered.^5^ Each of the RCTs has limitations that prevent their findings being translated into clinical practice. The limitations that are highlighted include the exclusion from study entry of patients with significant concerns relating to violence, study sample attrition and the confounding effects of providing enhanced care as part of the study design.^5,7^ Cohort studies provide an alternative means of assessing the outcomes of CTOs. In cohort studies, patients can act as their own controls and outcome measures are compared on and off CTOs. In general, studies of this type report that CTOs are associated with increased community care and medication and reduced hospital admissions, although these are not universal findings.^7,8^

Evidence to date does not help clinicians in deciding which patient groups most benefit from CTOs. Although the typical patient treated under a CTO has a diagnosis of schizophrenia or mood disorder,^9,10^ the full breadth of psychiatric diagnoses are eligible in most jurisdictions. It is possible that CTOs are associated with different clinical outcomes depending on patient diagnosis. In New Zealand, compulsory treatment of mental illness is administered under the Mental Health (Compulsory Assessment and Treatment) Act 1992.^11^ Compulsory in-patient and community treatment occurs if patients are ‘mentally disordered’, defined as an abnormal state of mind of such a degree that it poses serious dangers to self or others, or seriously diminishes the capacity for self-care.^11^ Patients are required to accept treatment in the community when on CTOs. However, active refusal of medication in the community may result in a psychiatric admission as opposed to being enforced in the community. We previously reported the effectiveness of CTOs in New Zealand using routinely collected data from large databases.^12^ CTOs were associated with increased community care and increased dispensing of psychiatric medication. CTOs were also associated with clinically significant reductions in admission frequency and admission length for patients with psychotic disorders. Diagnosis was categorised according to psychotic disorder status, as we were focused on evaluating the impact of a range of moderators (age, gender, ethnicity, sociodemographic deprivation and diagnosis) on outcome. The association between CTO status and admissions for psychotic disorders was a novel finding. However, our previous analyses did not examine the impact of diagnosis beyond that of a psychotic disorder. It is possible that other diagnostic groupings have different response signatures to CTOs. For this reason, we chose to expand our previous research and complete this analysis evaluating the impact of diagnosis on clinical outcomes during compulsory community treatment.

## Method

The authors assert that all procedures contributing to this work comply with the ethical standards of the relevant national and institutional committees on human experimentation and with the Helsinki Declaration of 1975, as revised in 2008. All procedures involving human participants/patients were approved by the Human Research Ethics Committee of the University of Otago (reference number HD19/076). This study analysed large databases. The data were received in anonymised form using unique identifiers. As a consequence, informed consent was not required. Full details of the source data-sets and rationale for outcome measures are provided in our parent paper.^12^ Key components are summarised below, along with details relating to diagnosis.

### Data-sets

The Programme for the Integration of Mental Health Data (PRIMHD) is the national mental health information collection service for the Ministry of Health (MoH), New Zealand. The PRIMHD data-set records service activity and outcomes for all individuals who receive treatment from public sector secondary healthcare and non-governmental organisation mental health and addiction services.^13^ PRIMHD data were requested from the MoH for all patients started on a CTO between 1 January 2009 and 31 December 2018. Data were requested in an anonymised form using a unique identifier and included the following:

- date and duration of the first CTO (section 29, Mental Health Act 1992) and subsequent CTOs
- demographic information
- DSM-IV principal diagnostic codes
- service use information, including admissions to psychiatric institutions, duration of admissions and out-patient contacts.

The Pharmaceutical Collection is a data warehouse containing the vast majority of dispensing data for New Zealand.^14^ Psychiatric medication dispensing was requested for patients identified by the unique identifiers provided in the PRIMHD sample. Medications were categorised using the online Pharmaceutical Schedule – November 2019 (a New Zealand database of medications subsidised by the government).

### Diagnosis

The PRIMHD database provided the DSM-IV primary diagnoses for patients placed under CTOs during the 10-year study period. Patients could receive multiple diagnoses owing to the potential for repeated contact with specialist mental health services (SMHS) over the study period. For this reason, organising principles were applied to categorise the diagnostic data. We did this using a hierarchical approach to create the following diagnostic groupings: dementia disorders; psychotic disorders; bipolar I disorder; other bipolar disorders; major depressive disorder; personality disorders; other diagnosis; no diagnosis. The DSM-IV codes and diagnoses included in each category are shown in the supplementary material available at https://doi.org/10.1192/bjo.2022.547.

### Primary outcome measure

As recommended by Rugkåsa et al,^7^ we chose the number of psychiatric in-patient admissions per year on a CTO compared with the number of psychiatric in-patient admissions/year off CTOs to be the primary outcome measure. Admissions are a proxy measure for the effectiveness of community treatment. Admissions were required to be of greater than 48 h to exclude brief admissions for administering depot antipsychotic medication because of refusal to accept treatment in the community. Patients cannot be restrained for medication in the community, so brief admissions may be required to administer medication despite unwellness not being present.

### Secondary outcomes

- The number of psychiatric admission days/year during a CTO compared with psychiatric admission days/year off CTOs.
- Number of community contacts during a CTO compared with contacts off CTOs. Community contacts included input from specialist mental health services and non-governmental organisations. Phone contacts, ‘did not attend’ appointments and care coordination phone calls were excluded.
- Rates of psychiatric medication dispensing during a CTO compared with rates of medication dispensing off CTOs.

### Statistical analysis

The study population according to diagnostic grouping is described using standard descriptive statistics. Multiple pairwise comparisons were used to examine between-diagnostic group differences.

The incidence of the key outcome measures for each of the diagnostic groups was calculated for the periods on and off CTOs. Individual data were aggregated according to CTO status. The grouped period for patients on CTOs was compared with the grouped period for patients off CTOs. This involved calculating the person-years represented for each individual on and off CTOs over the total study period. The total number of relevant outcome events both on and off CTOs for each individual were then summed to calculate the rates on and off CTOs. A rate ratio (RR) was calculated using these incidences by dividing the incidence of the outcome measure on CTOs with the corresponding figure off CTOs. An RR < 1 means that the outcome is less likely on CTOs and an RR > 1 means the outcome measure is more likely on CTOs. The 95% confidence intervals for the incidence and ratio estimates were calculated using the standard Poisson approximation.^15^ Significance was set at *P* < 0.05. SPSS version 27 for Windows was used for analysis.

## Results

In total, 14 726 patients were placed on a CTO at any time during the 10-year period between 1 January 2009 and 31 December 2018. The breakdown of the study population according to diagnosis was as follows: dementia disorders, 2.4% (*n* = 350); psychotic disorders, 56.6% (*n* = 8338); bipolar I disorder, 9.5% (*n* = 1393); other bipolar disorders, 1.4% (*n* = 212); major depressive disorder, 2.8% (*n* = 417); personality disorders, 0.7% (*n* = 106); ‘other diagnosis’, 5.1% (*n* = 757); and ‘no diagnosis’, 21.4% (*n* = 3153).

Table 1 reports the characteristics of the study population according to diagnosis. The dementia disorders group (mean age 46.7 years, s.d. = 22.7) was significantly older than the other diagnostic groups (*P* < 0.01 for all pairwise comparisons). The personality disorders group (mean age 28.9 years, s.d. = 13.9) was significantly younger than the other diagnostic groups (*P* < 0.01 for all pairwise comparisons) except for the ‘other diagnosis’ group (mean age 29.5 years, s.d. = 15.6, *P* = 0.70). The mean age for the overall sample was 35.2 years (s.d. = 16.0). Gender breakdown is shown in Table 1. Notably, for personality disorders, 84% were female. The psychotic disorders group was 38.4% Māori ethnicity, which was a higher proportion than in all other diagnostic groups (*P* < 0.01). In contrast, the major depressive disorder group was 17.0% Māori, a lower proportion than for the other diagnostic groups (*P* < 0.01). The mean number of CTOs for the diagnostic groups over the study period ranged from 1.9 (major depressive disorder) to 3.0 (psychotic disorders). The psychotic disorders group spent longer on compulsory treatment over the study period (median 631.5 days, IQR = 1546.0 days) than the other diagnostic groups (*P* < 0.01 for all pairwise comparisons). The major depressive disorder group spent less time on compulsory treatment (median 170.0 days, IQR = 220.0 days) over the study period than other diagnostic groups (*P* < 0.01 for all pairwise comparisons) except personality disorders (median 182.5 days IQR = 426 days, *P* = 0.15).

**Table 1:** Characteristics of the study population according to diagnosis

Diagnosis	Age, years (mean, s.d.)	Male, %	Māori, %	CTOs, *n*: mean (s.d.)	Total duration of compulsory treatment, days: median (IQR)
Dementia disorders	46.7 (22.7)	57.4	24.0	2.2 (2.0)	183.0 (521.0)
Psychotic disorders	33.4 (14.5)	64.9	38.4	3.3 (3.0)	631.5 (1546.0)
Bipolar 1 disorder	39.0 (15.7)	48.2	28.9	3.0 (3.0)	283.0 (659.0)
Other bipolar disorders	39.4 (16.3)	46.2	24.5	2.5 (3.7)	194.5 (516.0)
Major depressive disorder	37.9 (17.3)	38.6	17.0	1.9 (2.7)	170.0 (220.0)
Personality disorders	28.9 (13.9)	16.0	25.5	2.3 (1.7)	182.5 (426.0)
Other diagnosis	29.5 (15.6)	56.8	30.6	2.0 (1.8)	182.0 (387.0)
No diagnosis	37.9 (17.6)	57.1	31.3	2.4 (2.4)	308.0 (1039.0)

CTO, community treatment order.

Table 2 reports admission frequency/year, psychiatric admission days/year and community psychiatric contacts/year for each of the diagnostic groups according to CTO status and the RRs for these variables.

**Table 2:** Rate ratios of key outcomes according to diagnostic group

Diagnosis	Outcome variable	Outcome variable per year on CTOs	Outcome variable per year off CTOs	Rate ratio	95% CI	*P*
Dementia disorders	Admission frequency	1.06	0.73	1.45	1.31–1.59	<0.01
	Admission days	13.61	9.78	1.39	1.36–1.43	<0.01
	Community contacts	79.61	20.23	3.94	3.89–3.99	<0.01
Psychotic disorders	Admission frequency	1.00	1.22	0.82	0.81–0.83	<0.01
	Admission days	12.32	15.17	0.81	0.81–0.82	<0.01
	Community contacts	102.09	38.60	2.64	2.64–2.65	<0.01
Bipolar I disorder	Admission frequency	1.24	1.11	1.12	1.07–1.16	<0.01
	Admission days	12.98	10.29	1.26	1.25–1.28	<0.01
	Community contacts	83.73	26.79	3.13	3.11–3.14	<0.01
Other bipolar disorders	Admission frequency	1.06	0.95	1.12	1.00–1.27	0.05
	Admission days	12.88	10.06	1.28	1.24–1.32	<0.01
	Community contacts	89.94	31.12	2.89	2.85–2.93	<0.01
Major depressive disorder	Admission frequency	2.21	1.09	2.02	1.87–2.19	<0.01
	Admission days	26.23	11.87	2.21	2.16–2.26	<0.01
	Community contacts	90.26	25.38	3.56	3.51–3.60	<0.01
Personality disorders	Admission frequency	3.50	2.40	1.45	1.31–1.61	<0.01
	Admission days	32.88	24.02	1.37	1.32–1.41	<0.01
	Community contacts	113.16	52.96	2.14	2.10–2.18	<0.01
Other diagnosis	Admission frequency	1.19	0.84	1.41	1.32–1.50	<0.01
	Admission days	14.98	9.68	1.55	1.52–1.57	<0.01
	Community contacts	86.09	21.17	4.07	4.03–4.10	<0.01
No diagnosis	Admission frequency	0.87	0.78	1.11	1.08–1.14	<0.01
	Admission days	10.92	9.15	1.19	1.18–1.20	<0.01
	Community contacts	81.77	21.90	3.73	3.72–3.75	<0.01

### Admission frequency

**Fig. 1:**
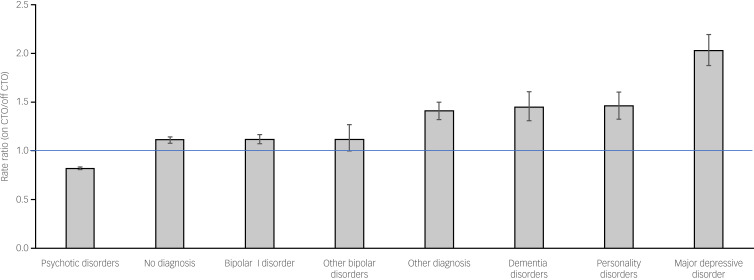
Rate ratio of admission frequency according to diagnostic group. Error bars represent 95% CIs; the horizontal line highlights RR = 1.

The psychotic disorders group was the only diagnostic group with less frequent admissions on CTOs (1.00 admissions/year on CTOs compared with 1.22 admissions/year off CTOs; RR = 0.82, 95% CI 0.81–0.83, *P* < 0.01). All other diagnostic groups had more admissions/year on CTOs (Table 2). The largest RR was for the major depressive disorder group (2.21 admissions/year on CTOs compared with 1.09 admissions/year off CTOs; RR = 2.02, 95% CI 1.87–2.19, *P* < 0.01). The personality disorders group were admitted more frequently than other diagnostic groups on and off CTOs, with a significantly higher rate on CTOs compared with off CTOs (3.50 admissions/year on CTOs compared with 2.40 admissions/year off CTOs; RR = 1.46, 95% CI 1.31–1.61, *P* < 0.01). Fig. 1 reports the RR for admission frequency according to diagnostic group in ascending order to demonstrate the variation between diagnostic groups for this outcome measure.

### Admission days/year

The psychotic disorders group was the only diagnostic group with fewer admission days/year on CTOs (12.32 days/year on CTOs compared with 15.17 days/year off CTOs; RR = 0.81, 95% CI 0.81–0.82, *P* < 0.01) (Table 1). All other diagnostic groups had more admission days/year on CTOs (Table 2). The major depressive disorder group had many more admission days/year on CTOs (26.23 days/year compared with 11.87 days/year off CTOs, RR = 2.21, 95% CI 2.16–2.26, *P* < 0.01). The personality disorders group had the greatest number of admission days/year of any diagnostic group (32.88 days/year on CTOs compared with 24.02 days/year off CTOs, RR = 1.37, 95% CI 1.32–1.41, *P* < 0.01).

### Contacts with community psychiatric services

The number of contacts with community psychiatric services was increased for all diagnostic groups on CTOs (Table 2). The lowest RR was for personality disorders (113.16 contacts/year on CTOs compared with 52.96 contacts/year off CTOs (RR = 2.14, 95% CI 2.10–2.18, *P* < 0.01). The highest RR was for the ‘other diagnosis’ group (86.09 contacts/year on CTOs compared with 21.17 contacts/year off CTOs (RR = 4.07, 95% CI 4.03–4.10, *P* < 0.01).

### Dispensing of psychiatric medication

Table 3 reports the dispensing of psychiatric medications according to CTO status and diagnostic group. Dispensing of all psychiatric medications was increased on CTOs. This association was most prominent for depot antipsychotic medications. The RR for depot antipsychotic dispensing ranged from 2.85 for the psychotic disorders group to 8.34 for the major depressive disorders group.

**Table 3:** Rate ratio of psychiatric medication dispensing according to diagnostic grouping

		Dispensing rate/year on CTO	Dispensing rate/year off CTO	Rate ratio	95% CI	*P*
Dementia disorders	Antidepressants	4.41	3.34	1.32	1.26–1.39	<0.01
	Anxiolytics	4.37	1.82	2.40	2.28–2.52	<0.01
	Depot antipsychotics	5.58	1.05	5.29	5.03–5.56	<0.01
	Oral antipsychotics	30.06	10.92	2.75	2.70–2.81	<0.01
	Sodium valproate	4.27	1.05	4.06	3.8–4.29	<0.01
Psychotic disorders	Antidepressants	5.85	3.79	1.55	1.54–1.56	<0.01
	Anxiolytics	6.07	3.09	1.96	1.95–1.98	<0.01
	Depot antipsychotics	6.14	2.16	2.85	2.83–2.87	<0.01
	Oral antipsychotics	43.60	19.51	2.23	2.23–2.24	<0.01
	Sodium valproate	7.16	2.89	2.48	2.46–2.49	<0.01
Bipolar I disorder	Antidepressants	4.93	4.18	1.18	1.16–1.20	<0.01
	Anxiolytics	6.05	3.23	1.87	1.84–1.91	<0.01
	Depot antipsychotics	4.71	1.10	4.27	4.16–4.38	<0.01
	Oral antipsychotics	42.51	19.10	2.23	2.21–2.24	<0.01
	Sodium valproate	17.00	6.27	2.71	2.68–2.75	<0.01
Other bipolar disorders	Antidepressants	5.79	5.51	1.05	1.00–1.10	0.05
	Anxiolytics	7.46	6.39	1.17	1.12–1.22	<0.01
	Depot antipsychotics	4.56	0.95	4.82	4.49–5.18	<0.01
	Oral antipsychotics	32.64	19.63	1.66	1.63–1.70	<0.01
	Sodium valproate	13.58	5.80	2.34	2.26–2.43	<0.01
Major depressive disorders	Antidepressants	39.30	16.90	2.33	2.28–2.37	<0.01
	Anxiolytics	8.83	5.23	1.69	1.62–1.75	<0.01
	Depot antipsychotics	2.30	0.28	8.34	7.59–9.15	<0.01
	Oral antipsychotics	48.01	16.75	2.87	2.82–2.92	<0.01
	Sodium valproate	1.92	1.01	1.90	1.75–2.07	<0.01
Personality disorders	Antidepressants	57.61	26.32	2.19	2.13–2.25	<0.01
	Anxiolytics	46.98	18.96	2.48	2.41–2.55	<0.01
	Depot antipsychotics	3.30	0.87	3.80	3.37–4.27	<0.01
	Oral antipsychotics	78.74	44.61	1.76	1.73–1.80	<0.01
	Sodium valproate	10.67	6.21	1.72	1.62–1.82	<0.01
Other diagnosis	Antidepressants	15.59	7.93	1.97	1.93–2.00	<0.01
	Anxiolytics	9.88	5.65	1.75	1.71–1.79	<0.01
	Depot antipsychotics	6.06	0.84	7.25	6.99–7.53	<0.01
	Oral antipsychotics	37.92	13.40	2.83	2.80–2.86	<0.01
	Sodium valproate	5.52	1.67	3.30	3.19–3.41	<0.01
No diagnosis	Antidepressants	6.09	3.17	1.92	1.90–1.94	<0.01
	Anxiolytics	4.53	2.00	2.27	2.24–2.30	<0.01
	Depot antipsychotics	5.74	1.07	5.37	5.28–5.45	<0.01
	Oral antipsychotics	36.04	12.71	2.84	2.82–2.85	<0.01
	Sodium valproate	8.15	2.61	3.12	3.09–3.16	<0.01

## Discussion

We analysed data for all New Zealanders placed on a CTO over a 10-year period and report that there are differing outcomes according to diagnosis. We therefore believe that a more nuanced understanding of outcomes associated with CTOs is required. Our findings direct attention to the likely outcomes for people with a range of diagnoses on CTOs and whether appropriate clinical goals are being achieved with CTO use.

This study repeated our previous finding relating to CTO outcomes for people with psychotic disorders.^12^ A psychotic disorder diagnosis was associated with 22% fewer admissions on CTOs and shorter admission days by 2.85 days/year on CTOs. We regard the extent of these reductions in admissions to be clinically significant for patients and services. We suspect that the reduction in admissions results from greater use and adherence to antipsychotic medications. CTOs can therefore be regarded as meeting one of their goals of reducing ‘revolving door’ admissions for this patient group. This finding can be incorporated into evidence-informed discussions between clinicians, patients and family.

No other patient diagnostic group was associated with reduced admission frequency or admission days/year. Despite more contact with community out-patient services and higher rates of psychiatric medication, including depot antipsychotic medications, admission frequency and admission days/year for the other diagnostic groups were increased on CTOs. This finding requires close attention, as a key goal of reducing admissions is not being met.

The bipolar disorder groups had 12% more admissions on CTOs and more than 2 extra days in hospital/year on CTOs. They were also four to five times more likely to be dispensed depot antipsychotic medications. Atypical antipsychotic medications, including risperidone in depot form, are indicated for bipolar disorder and the prevention of mania (although clinical trials have not included non-consenting patients).^16,17,18^ Despite this evidence base, their increased use while patients were on CTOs was not associated with less frequent admissions compared with voluntary periods. It appears that increased care and higher dispensing rates for psychiatric medications (including depot antipsychotic medications) were ineffective in sufficiently modifying the course of illness to reduce admissions.

Patients with major depressive disorder were twice as likely to be admitted and had many more admission days/year on CTOs. In addition, the major depressive disorder group was eight times more likely to be dispensed depot antipsychotic medications on CTOs. Although augmentation with second-generation antipsychotics is an option for treatment-resistant depression,^19^ depot antipsychotic medications are not usually recommended for the treatment of major depressive disorder.^18^ The extent of the increase in depot antipsychotic dispensing is therefore striking. We assume the major depressive disorder group included patients with treatment-resistant depression and psychotic features. However, the increase in psychiatric medication on CTOs did not modify the course of illness sufficiently to reduce admissions compared with voluntary periods.

The core deficit in dementia disorders is a decline in cognitive function.^20^ Individuals with a dementia disorder diagnosis treated under a CTO are more likely to be impaired in their decision-making capacity than those with other disorders. In the present study, antipsychotics and sodium valproate were prescribed significantly more while these patients were on CTOs. They were presumably used to treat the behavioural and psychological symptoms of dementia, although evidence for their effectiveness is limited and there are concerns about their side-effect profile.^21,22^ Again, the higher rates of medication did not modify the course of illness sufficiently to reduce admissions compared with voluntary periods.

The personality disorders group had more frequent admissions on CTOs. Their admission days/year were greater than those for the other diagnostic groups and lengthened further in association with CTOs. Participants with personality disorders were dispensed medications, including depot antipsychotic medication, at much higher rates on CTOs. Treatment guidelines for personality disorders suggest only a limited role for medications.^23,24^ In addition, it is generally accepted that longer admissions for patients with personality disorders may be harmful and treatment should largely occur in the community.^25^ Although clinicians may argue that the complexity of presentation of patients with personality disorders should not preclude treatment under a CTO, our findings suggest that CTOs for personality disorders are associated with more frequent admissions and high rates of compulsory medication. The personality disorders group also received higher rates of community contacts on and off CTOs compared with the other diagnostic groups, suggesting that more intensive management is required for this group than for the other diagnostic categories.

Diagnoses in the ‘other diagnosis’ group included post-traumatic stress disorder, alcohol dependence, opioid dependence, cannabis dependence, and substance induced mood and psychotic disorders (despite the New Zealand Mental Health Act excluding substance use as a basis for providing compulsory psychiatric treatment). This group experienced more frequent admissions, more admission days/year, more community care and greater dispensing of psychiatric medication, including depot antipsychotic medications, on CTOs. Similarly to the other non-psychotic disorder groups, higher rates of treatment on CTOs did not reduce admissions compared with voluntary periods.

We chose admission frequency (excluding brief ‘recall admissions’) to be the primary outcome measure as recommended by Rugkåsa et al.^7^ This does not mean we regard admissions in a negative light. We believe them to be an essential part of psychiatric management and often the only solution if community care is progressing poorly. However, we used admissions as a crude proxy for relapse and categorical measure of CTO ‘success’. On this basis, should increased admissions and more admission days/year for the non-psychotic disorder groups be regarded as a failure of CTOs? At first glance, it could be argued that CTOs should not be used for non-psychotic disorder groups because compulsory treatment (including high rates of depot antipsychotic medications) does not result in reduced admissions compared with periods off CTOs.

However, this was not an RCT. Greater degrees of unwellness are likely to be clustered around the times CTOs are used. Increased admissions may therefore simply mark periods of worse mental health which clinicians attempt to contain by using CTOs for individuals who are reluctant to take medication. In this situation, unless the medication is rapidly and reliably effective, it is perhaps not surprising that admissions are more frequent.

For psychotic disorders, compulsory treatment appears effective and reduces admissions, although higher rates of medication use and more community care will not be viewed favourably by all and some will argue that involuntary medication use and care in the community remains unjustified despite apparent clinical benefits. For the non-psychotic disorder groups, compulsory treatments are either less effective or less quickly effective, so that admissions are not reduced on CTOs. The psychotic disorder group in our study spent longer on CTOs than the other groups. Our data do not inform us whether longer periods of compulsory treatment would eventually result in reduced admissions compared with voluntary periods for non-psychotic disorder groups. However, the association between high admission frequency accompanied by high rates of depot antipsychotic medications and community care is a concern in situations where there is not an obvious indication for their use (or if their use does not achieve demonstrable clinical success). We believe that coercive treatments should not be used lightly and their use should be justifiable to others. For patients, compulsory community treatment is accompanied by feelings of coercion and control.^26^ It is possible that disruption to therapeutic relationships contributes to the lack of treatment efficacy. We therefore recommend that the associations we report for non-psychotic disorders be considered by clinicians and compulsory treatment be reviewed in light of our findings.

The psychotic disorders group had a higher percentage of Māori patients (38.4%) and the depressive disorders group had a lower percentage of Māori patients (17.0%) than the other diagnostic groups. Several factors are likely to affect these findings, including high burden of mental illness among Māori and differential access to treatment.^27,28^ Our study could not inform us whether systemic biases also contributed to the ethnic variation observed between diagnoses. We anticipate a further paper scrutinising CTOs for Māori more closely.

### Limitations

Our study utilised routinely collected data. Unfortunately, symptom or functional outcome scales were not included in the data-set. Although admissions and medication dispensing are readily quantifiable, community care was defined broadly and the quality as opposed to quantity of community interventions provided is unknown. The study was strengthened by the inclusion of all individuals placed on CTOs over the study period. However, individuals were not randomised to CTOs; instead, these were recommended by clinicians and endorsed by judges at CTO hearings. Non-random elements are therefore inherent in the distribution between on CTO/off CTO status. For example, CTOs are more likely to be introduced during times when there is heightened concern about illness and risk, and ceased at other times. Any reduction in admission frequency on CTOs therefore represents a clinical benefit during times when there are greater concerns (as was observed for psychotic disorders). For the diagnostic groups that did not have reduced admission frequency on CTOs, it is possible that admission rates would have been still higher in the absence of a CTO at that time. However, it is clear that the compulsory interventions did not reduce rates compared with the non-compulsory period.

Patients in the ‘no diagnosis’ group comprised 21.3% of the study population. It would be surprising if the majority of these patients were not given clinical diagnoses over the study period. We therefore expect that the absence of PRIMHD diagnoses relates to reporting problems acknowledged to be present in the PRIMHD database.^29^ Despite this limitation, we regard the positively identified categories to be a sensitive representation of the clinical issues for the individuals represented.

The dementia disorders group had a mean age of 46.7 years. This is younger than expected for a typical dementia cohort. However, in New Zealand, compulsory treatment for dementia is usually provided under a capacity-based legislation called the Protection of Personal and Property Rights Act 1988.^30^ This enables broader interventions (including financial and physical healthcare) to be applied, unlike the New Zealand Mental Health Act (which only enables compulsory treatment of mental disorder). Consequently, the dementia group in our study are likely to be a heterogeneous group mostly consisting of individuals with pre-senile dementias treated and managed in adult as opposed to older aged services.

We applied hierarchical rules to the diagnostic database to stratify large data into a form that could be analysed according to diagnostic groupings. Readers familiar with DSM-III^31^ will be aware that this approach is not without precedent, although more recent iterations of the DSM have largely removed the hierarchical approach to diagnosis. We believe that the application of hierarchical rules in this analysis is a sensible and informative way of managing complex information, although we recognise that comorbidity is common and that clinicians will need to translate our findings to inform their approach to complex clinical scenarios.

Our study was situated in New Zealand. The New Zealand Mental Health Act^11^ applies risk-based criteria to the threshold for compulsory treatment. We think it likely that the CTO signatures observed in our study will be present in countries with similar legislation and recommend other researchers review existing databases to clarify whether our findings are reproduced elsewhere.

### Clinical implications

There are specific outcome signatures relating to CTOs according to diagnosis. Rather than asking whether CTOs are effective, we believe that awareness of the likely outcomes for different diagnostic groupings will assist clinicians in making informed decisions about CTOs and in communicating their thinking to patients and families. We believe our research remains supportive of CTOs for patients with psychotic disorders. However, we believe our findings should challenge clinicians to review the use of CTOs for patients without psychotic disorders. The clustering of higher dispensing of psychiatric medications, including depot antipsychotics, alongside more frequent and longer admissions on CTOs is concerning. Although more frequent admissions on CTOs may be a necessary response to difficult clinical scenarios, we believe that an explicit awareness of likely outcomes for different diagnoses has the potential to improve clinical practice in this complex area.

## Data Availability

On reasonable request the corresponding author, B.B., will support access to the aggregated data-set from which this study is drawn.

## Supplementary material

For supplementary material accompanying this paper visit http://doi.org/10.1192/bjo.2022.547. click here to view supplementary material

## References

[1] Callaghan S, Newton-Howes G. Coercive community treatment in mental health: an idea whose time has passed? J Law Med 2017; 24: 900–14.

[2] Swartz MS, Swanson JW, Wagner HR, Burns BJ, Hiday VA, Borum R. Can involuntary outpatient commitment reduce hospital recidivism? Findings from a randomized trial with severely mentally ill individuals. Am J Psychiatry 1999; 156: 1968–75. 10588412 10.1176/ajp.156.12.1968

[3] Steadman HJ, Gounis K, Dennis D, Hopper K, Roche B, Swartz M, Assessing the New York City involuntary outpatient commitment pilot program. Psychiatr Serv 2001; 52: 330–6. 11239100 10.1176/appi.ps.52.3.330

[4] Burns T, Rugkåsa J, Molodynski A, Dawson J, Yeeles K, Vazquez-Montes M, Community treatment orders for patients with psychosis (OCTET): a randomised controlled trial. Lancet 2013; 381: 1627–33. 23537605 10.1016/S0140-6736(13)60107-5

[5] Kisely SR, Campbell LA, O'Reilly R. Compulsory community and involuntary outpatient treatment for people with severe mental disorders. Cochrane Database Syst Rev 2017; 3: CD004408. 28303578 10.1002/14651858.CD004408.pub5 PMC6464695

[6] Swartz MS, Wilder CM, Swanson JW, Van Dorn RA, Robbins PC, Steadman HJ, Assessing outcomes for consumers in New York's assisted outpatient treatment program. Psychiatr Serv 2010; 61: 976–81. 20889634 10.1176/ps.2010.61.10.976

[7] Rugkåsa J, Dawson J, Burns T. CTOs: what is the state of the evidence? Soc Psychiatry Psychiatr Epidemiol 2014; 49: 1861–71. 24562319 10.1007/s00127-014-0839-7

[8] Kisely SR, Xiao J, Preston NJ. Impact of compulsory community treatment on admission rates: survival analysis using linked mental health and offender databases. Br J Psychiatry 2004; 184: 432–8. 15123508 10.1192/bjp.184.5.432

[9] Barkhuizen W, Cullen AE, Shetty H, Pritchard M, Stewart R, McGuire P, Community treatment orders and associations with readmission rates and duration of psychiatric hospital admission: a controlled electronic case register study. BMJ Open 2020; 10(3): e035121. 10.1136/bmjopen-2019-035121 PMC7059496 32139493

[10] Dey S, Mellsop G, Obertova Z, Jenkins M. Sociodemographic and clinical variables associated with discharge under compulsory treatment orders. Australas Psychiatry 2021; 29: 163–8. 33354991 10.1177/1039856220970054

[11] New Zealand Parliamentary Counsel Office/Te Tari Tohutohu Pāremata. Mental Health (Compulsory Assessment and Treatment) Act 1992. New Zealand Government, 1992 (http://www.legislation.govt.nz/act/public/1992/0046/latest/whole.html).

[12] Beaglehole B, Newton-Howes G, Frampton C. Compulsory community treatment orders in New Zealand and the provision of care: an examination of national databases and predictors of outcome. Lancet Reg Health West Pac 2021; 17: 100275. 34734198 10.1016/j.lanwpc.2021.100275 PMC8488594

[13] Ministry of Health (Manatū Hauora). PRIMHD – Mental Health Data Wellington. New Zealand Government, 2016 (http://www.health.govt.nz/nz-health-statistics/national-collections-and-surveys/collections/primhd-mental-health-data).

[14] Ministry of Health (Manatū Hauora). Pharmaceutical Collection. New Zealand Government, 2019 (https://www.health.govt.nz/nz-health-statistics/national-collections-and-surveys/collections/pharmaceutical-collection).

[15] Sahai H, Khurshid A. Formulas and tables for the determination of sample sizes and power in clinical trials for testing differences in proportions for the matched pair design: a review. Fundam Clin Pharmacol 1996; 10: 554–63. 8985726 10.1111/j.1472-8206.1996.tb00614.x

[16] Boyce P, Irwin L, Morris G, Hamilton A, Mulder R, Malhi GS, Long-acting injectable antipsychotics as maintenance treatments for bipolar disorder: a critical review of the evidence. Bipolar Disord 2018; 20(suppl 2): 25–36. 30328222 10.1111/bdi.12698

[17] Pacchiarotti I, Tiihonen J, Kotzalidis GD, Verdolini N, Murru A, Goikolea JM, Long-acting injectable antipsychotics (LAIs) for maintenance treatment of bipolar and schizoaffective disorders: a systematic review. Eur Psychopharmacol 2019; 29: 457–70. 10.1016/j.euroneuro.2019.02.003 30770235

[18] Malhi GS, Bell E, Singh AB, Bassett D, Berk M, Boyce P, The 2020 Royal Australian and New Zealand College of Psychiatrists clinical practice guidelines for mood disorders: major depression summary. Bipolar Disord 2020; 22: 788–804. 33320412 10.1111/bdi.13035

[19] Mulder R, Hamilton A, Irwin L, Boyce P, Morris G, Porter RJ, Treating depression with adjunctive antipsychotics. Bipolar Disord 2018; 20(suppl 2): 17–24. 30328223 10.1111/bdi.12701

[20] American Psychiatric Association. Diagnostic and Statistical Manual of Mental Disorders (5th edn) (DSM-5). American Psychiatric Publishing, 2013.

[21] Tible OP, Riese F, Savaskan E, von Gunten A. Best practice in the management of behavioural and psychological symptoms of dementia. Ther Adv Neurol Disord 2017; 10: 297–309. 28781611 10.1177/1756285617712979 PMC5518961

[22] Lee PE, Gill SS, Freedman M, Bronskill SE, Hillmer MP, Rochon PA. Atypical antipsychotic drugs in the treatment of behavioural and psychological symptoms of dementia: systematic review. BMJ 2004; 329: 75. 15194601 10.1136/bmj.38125.465579.55 PMC449807

[23] Ripoll LH, Triebwasser J, Siever LJ. Evidence-based pharmacotherapy for personality disorders. Int J Neuropsychopharmacol 2011; 14: 1257–88. 21320390 10.1017/S1461145711000071

[24] National Collaborating Centre for Mental Health. Borderline Personality Disorder: Treatment and Management (NICE Clinical Guideline CG78). National Institute for Health and Care Excellence, 2009. 39480982

[25] Paris J. Is hospitalization useful for suicidal patients with borderline personality disorder? J Pers Disord 2004; 18: 240–7. 15237044 10.1521/pedi.18.3.240.35443

[26] Corring D, O'Reilly R, Sommerdyk C. A systematic review of the views and experiences of subjects of community treatment orders. Int J Law Psychiatry 2017; 52: 74–80. 28325533 10.1016/j.ijlp.2017.03.002

[27] Baxter J, Kokaua J, Wells JE, McGee MA, Oakley Browne MA, New Zealand Mental Health Survey Research Team. Ethnic comparisons of the 12 month prevalence of mental disorders and treatment contact in Te Rau Hinengaro: the New Zealand Mental Health Survey. Aust N Z J Psychiatry 2006; 40: 905–13. 16959017 10.1080/j.1440-1614.2006.01910.x

[28] Metcalfe S, Beyene K, Urlich J, Jones R, Proffitt C, Harrison J, Te Wero tonu – the challenge continues: Māori access to medicines 2006/07–2012/13 update. N Z Med J 2018; 131: 27–47. 30408816

[29] Ministry of Health. PRIMHD Classification – Summary and Metadata (version 1.3 updated 13/03/2018). Ministry of Health, 2018 (https://www.health.govt.nz/system/files/documents/pages/primhd_-_classification_metadata.docx).

[30] New Zealand Parliamentary Counsel Office/Te Tari Tohutohu Pāremata. Protection of Personal and Property Rights Act 1988. New Zealand Government, 1988 (https://www.legislation.govt.nz/act/public/1988/0004/latest/whole.html).

[31] American Psychiatric Association. Diagnostic and Statistical Manual of Mental Disorders (3rd edn) (DSM-III). American Psychiatric Association, 1980.

