# Supplementary material for: Impact of diagnosis on outcomes for compulsory treatment orders in New Zealand
Source: BJPsych Open. 2022 Aug 1;8(5):e145. doi: 10.1192/bjo.2022.547 (PMC9380042; doi:10.1192/bjo.2022.547)
Supplement: Supplementary file 1 [file S2056472422005476sup001.docx]

**Supplement 1**

Organising principles for the diagnostic groupings

1. Dementia Disorders. Their DSM IV codes are 2900, 29010, 29011, 29012, 29013, 29020, 29021, 2903, 29040, 29041, 29042, 29043, 2910, 2911, 2912, 2940, 2941, 2948, 7809. These codes correspond to Dementias of Alzheimers type, Dementia due to Pick’s Disease, Dementia due to Creutzfeld-Jacob Disease, Vascular Dementia, Alcohol-Induced Amnestic Disorder and Dementia, Amnestic Disorder (due to a General Medical Condition), Dementia due to HIV, a General Medical Condition, Dementia NOS, Age Related Cognitive Decline
2. Psychotic Disorder. This includes anybody with the following DSM IV codes who does not also have a Dementia disorder. The clinical codes are 29510, 29520, 29530, 29540, 29560, 29570, 29590, 2971, 2973, 2988, and 2989. These codes relate to Schizophrenia and subtypes, Schizoaffective Disorder, Delusional Disorder, Shared Psychotic Disorder, Brief Psychotic Disorder, Schizophreniform Disorder, Psychotic Disorder NOS.
3. Bipolar 1 Disorder. This includes anyone with the following DSM IV codes who does not also have a diagnosis reported above. The codes are 29600, 29601, 29602, 29603, 29604, 29605, 29606, 29640, 29641, 29642, 29643, 29644, 29645, 29646, 29650, 29651, 29652, 29653, 29654, 29655, 29656, 29660, 29661, 29662, 29663, 29664, 29665, 29666, 2967. These codes relate to Bipolar 1 Disorder diagnoses (with or without Psychotic features)
4. Other Bipolar Disorders. This includes anyone with the following DSM IV codes who does not also have a diagnosis reported above. The codes are 29689 and 29680. These codes relate to Bipolar 2 Disorder and Bipolar Disorder NOS.
5. Major Depressive Disorder. This includes anyone with the following DSM IV codes who does not also have a diagnosis reported above. The codes are 29620, 29621, 29622, 29623, 29624, 29625, 29626, 29630, 29631, 29632, 29633, 29634, 29635, 29636. These codes relate to various Major Depressive Disorder diagnoses.
6. Personality Disorders. This includes anyone with the following DSM IV codes who does not have a diagnosis reported above. The codes are 3010, 30120, 3014, 30150, 3016, 3017, 30181, 30182, 30183, 3019. These codes relate to a range of Personality disorder diagnoses.
7. Other diagnosis: Patients given a DSM IV coded diagnosis and not represented above. Diagnoses contained within this group include Post-Traumatic Stress Disorder, Alcohol Dependence, Opioid Dependence, Cannabis Dependence, and Substance Induced Mood and Psychotic Disorders (despite the New Zealand Mental Health Act excluding substance use as a basis for providing compulsory psychiatric treatment).
8. No diagnosis: This group contains codes 7999 (Diagnosis or Condition Deferred on Axis I or Diagnosis Deferred on Axis II), V7109 (No Diagnosis or Condition on Axis I or Axis II), and patients who were placed on a CTO but did not have a diagnosis recorded in PRIMHD during the study period.
